# Finite Difference Interpolation for Reduction of Grid-Related Errors in Real-Space Pseudopotential Density Functional Theory

**DOI:** 10.1021/acs.jctc.3c00217

**Published:** 2023-06-29

**Authors:** Deena Roller, Andrew M. Rappe, Leeor Kronik, Olle Hellman

**Affiliations:** † Weizmann Institute of Science, Department of Molecular Chemistry and Materials Science, Weizmann Institute of Science, Rehovoth 76100, Israel; ‡ Department of Chemistry, University of Pennsylvania, Philadelphia, Pennsylvania 19104, United States

## Abstract

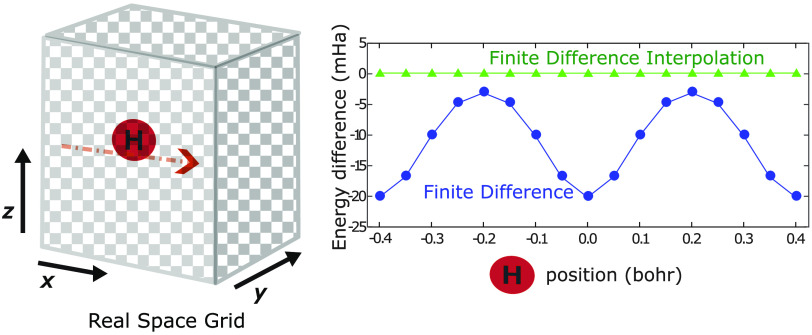

The real-space pseudopotential approach is a well-known method for large-scale density functional theory (DFT) calculations. One of its main limitations, however, is the introduction of errors associated with the positioning of the underlying real-space grid, a phenomenon usually known as the “egg-box” effect. The effect can be controlled by using a finer grid, but this raises the cost of the calculations or even undermines their feasibility altogether. Therefore, there is ongoing interest in the reduction of the effect per a given real-space grid. Here, we present a finite difference interpolation of electron orbitals as a means of exploiting the high resolution of the pseudopotential to reduce egg-box effects systematically. We implement the method in PARSEC, a finite difference real-space pseudopotential DFT code, and demonstrate error mitigation and improved convergence at a low additional computational cost.

## 1 Introduction

Density functional theory (DFT) is a well-known approach for first-principles calculations.^1−3^ It has become a standard tool in many areas of physics, chemistry, and materials science research.^4−7^ Most standard codes employed for solving the equations associated with DFT rely on localized atomic orbitals^8−15^ or plane waves.^16−24^ However, real-space methods have long been of interest as a means of harnessing the power of massively parallel computers for the computation of ever larger systems of interest.^25−42^

Here, we focus on a specific real-space approach, known as the finite-difference pseudopotential method,^25^ in which all quantities of interest are sampled on a real-space grid. The Laplacian associated with the kinetic energy is then treated using a high-order finite difference operator, and core electrons are suppressed via the use of pseudopotentials.^43^ This approach results in a sparse Hamiltonian that is amenable to massively parallel computing, and has already been used for calculations with up to tens of thousands of atoms.^44^

A major disadvantage of the finite difference real-space approach is associated with the aliasing of grid-based quantities owing to insufficient sampling of rapidly varying functions. Because the aliasing error depends on the placement of the atoms relative to the underlying grid, continuously translating (or rotating) the system relative to the sampling grid results in an oscillatory error, usually known as the “egg-box” or “egg-carton” error.^8,45^ This error can be highly problematic for geometry sensitive DFT calculations, e.g., geometric relaxation, molecular dynamics simulations, computation of vibrational data, and more. Clearly, egg-box errors can always be reduced by increasing the spatial sampling rate, i.e., making the grid finer. However, this results in a power-law escalation of the computational cost, which often hinders the calculation due to computer time and/or memory issues. Reducing egg-box errors at a tolerable computational cost is therefore an ongoing challenge.

In prior work, the reduction of egg-box effects has been mainly investigated from three distinct perspectives: one which addresses the grid, one which addresses the pseudopotential, and a third which addresses the representation of operators on the grid. Grid-oriented approaches mostly rely on the introduction of additional denser grids near atomic nuclei, where the pseudopotential is rapidly varying, thereby avoiding the high cost of using a finer grid everywhere. This can be achieved via a multigrid^28,46−48^ or an adaptive grid^34,49^ approach. While certainly useful, such approaches can be cumbersome to implement, increase the computational cost, and may adversely affect parallelization. Pseudopotential-oriented methods therefore attempt to alleviate the problem by altering the pseudopotentials such that all grid-related quantities become smoother and aliasing errors are automatically reduced. One example of such approaches is the filtering of existing pseudopotentials,^8,50,51^ while compensating for the lost charge. Another example is the adoption of smoother pseudopotentials^52,53^ (originally developed in the context of planewave codes) or the use of alternative methods such as the projector augmented waves in real-space.^40^ Such approaches are undoubtedly useful, but in the end pseudopotentials cannot be smoothed indefinitely and the problem remains, especially for certain classes of atoms, e.g., first-row atoms and transition-metal atoms.

The third approach modifies neither the grid nor the pseudopotential but instead tries to solve the underlying equations with higher accuracy by changing the representation of the differential equations on the grid. A naive solution is to calculate the system at different placements on the grid, yielding an averaged solution. This will reduce the egg-box error, but is inefficient and is not guaranteed to converge to the correct solution. Studies targeting the (nonlocal) force expression showed significant improvement in real-space simulations.^34,54,55^ Recently, Qiu et al. introduced the shift operator method.^56^ They proposed the construction of nonlocal potential operators based on a weighted sum of translations of the potential. This approach has shown significant mitigation of egg-box errors in model calculations. However, this may come at the cost of some numerical precision, and generalization to 3-D calculations at a low computational cost is challenging.

Inspired by the recent developments in grid representation methods, we present finite difference interpolation (FDI) for off-grid points as a higher accuracy representation of functions on a given grid. FDI is based on known basic finite difference limits^57^ and generalizes previous work^54^ to all operators. We show that this method follows the sparsity pattern of standard finite difference calculations, thus adding minimal cost and not disrupting massive parallelization. The article is arranged as follows: in section 2 we discuss the finite difference method and develop the FDI formalism. In section 3 we show results of FDI applied to a 1D model system and to pseudopotential DFT calculations, including an analysis of computational cost. Finally, in section 4 we present concluding remarks and an outlook.

## 2 Method and Implementation

In the finite difference approach, functions are represented by a vector of their values on a discrete grid and operators are represented by matrices. A general (nonmultiplicative) operator is represented by a general matrix, a local (multiplicative) operator is represented by a diagonal matrix, and an operator is referred to as semilocal if it is represented by a diagonally dominant sparse matrix. The application of an operator to a function consists of a matrix-vector multiplication. Therefore, computation and parallelization efficiency rely on the attainment of operators that are as sparse as possible yet still converge rapidly with grid size.

Finite difference approaches differ from other real-space methods, e.g., B-splines, finite elements, or real-space basis set methods, in that they do not utilize an explicit basis set. When using, e.g., B-splines or finite elements, there is a clear definition as to what value a function would take between two discrete grid points. In the finite difference approach, however, each function segment is represented by a single value, with no obvious recipe for what value a function would take in between grid points. To address this issue, we revisit the finite difference derivative operator.^58^

To find the derivative in the *x* direction, at grid point *k* located at *x*_*k*_, we can express the function values at neighboring grid points via a Taylor expansion. The Taylor expansion of order 2*n* at the *j*^th^ neighbor is given by
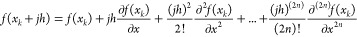
1where *h* is the grid spacing. As shown in detail in Appendix A, using *n* neighbors on each side of *x*_*k*_ we can set up 2*n* + 1 equations for the derivatives at *x*_*k*_, such that the derivatives , with *i* = 0, 1, 2, ..., 2*n* can be expressed in terms of function values at neighboring points:
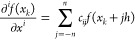
2where *c*_*ij*_ is the coefficient for the *i*^th^ derivative from the *j*^th^ neighbor. As further shown in Appendix A, the *c*_*ij*_ coefficients can also be found in terms of the inverse of a relevant Vandermonde (design) matrix. The expansion order used in practice determines a balance between the derivative precision on the one hand and the computational cost of derivative evaluation on the other hand.

We now use the high order finite difference derivatives to obtain a continuous representation of a discrete function. In eq 1 we used a Taylor series at the grid points. The same approach can be used to interpolate function values at points that are not necessarily placed on the grid:
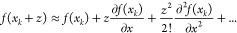
3where *x*_*k*_ + *z* is a position removed by  from the grid point *x*_*k*_. Here the high-order finite difference derivatives defined in eq 2 are used to approximate the derivatives. To express eq 3 as a matrix vector multiplication, we first define a selection operator, **L**_*k*_, which selects the neighboring points of grid point *k* that are relevant for the high order finite difference expansion around that point:
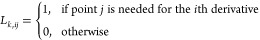
4The selection operator results in a “short” vector with *N*_stc_ nonzero entries per grid point *k*, where *N*_stc_ is the number of points used for the finite difference derivatives. We then collect the powers of *z* from eq 3 in a row vector:
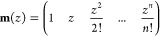
5and arrange the high order finite-difference derivative coefficients as introduced in eq 2 in matrix form, such that the row index corresponds to the derivative order and the column index designates the relative position of the neighbor points in terms of number of grid steps:
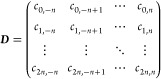
6Combining these operators, we express eq 3 as
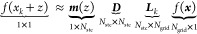
7where we have denoted the dimensions of the matrices for clarity. Here, *f*(***x***) denotes the vector of function values at *all* grid points. In essence, applying **L**_*k*_ selects the correct neighbors for grid point *k*, applying **D** returns the derivatives at *k*, and finally **m**(*z*) evaluates the function at point *z*.

**Figure 1:**
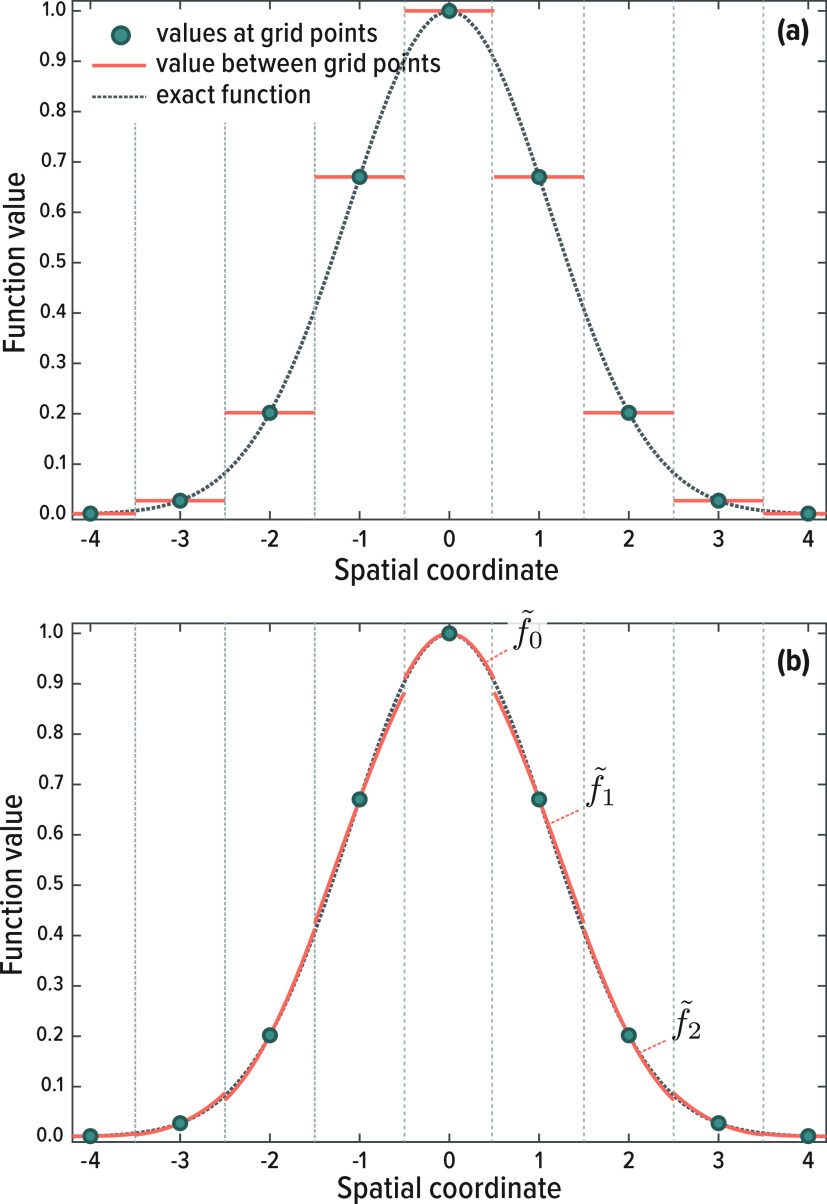
Illustration of the finite difference interpolation scheme introduced in this paper. As a test function we use a Gaussian and a grid spacing of 1. In panel (a) the function is approximated as fixed around grid points. In panel (b) eq 7 is used to estimate the value around the grid points as piecewise polynomials. In this example we used a stencil that includes two grid points in each direction, yielding a fifth-order polynomial.

For a more general notation we define the continuously interpolated function around the grid point *x*_*k*_ as
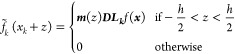
8Contributions from all grid points are then combined to define the full interpolated function, *f*_I_(*x*), across the entire simulation domain.
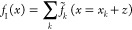
9where *x* is a continuous variable (in contrast to *x*_*k*_ which is discrete). At any specific value of *x*, only a single *f̃*_*k*_ will be nonzero (i.e., *f*_I_(*x*) takes on the value of the Taylor expansion from the closest point). Figure 1 demonstrates the interpolation scheme by applying it to a test function. Note that in panel B the interpolation polynomials are not continuous. One could enforce continuity of the polynomials, thus creating a spline interpolation. However, by using the piecewise polynomial we retain a grid based, sparse representation of the Hamiltonian.

The representation in eq 9 allows us to define the discrete counterpart to any operator in a manner consistent with FD. The expectation value for an operator *Ô* is
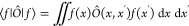
10If we then replace the continuous function *f*(*x*) with *f*_I_(*x*) of eq 9, we obtain
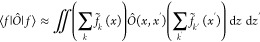
11where *x* = *x*_*k*_ + *z*, *x*′ = *x*_*k*′_ + *z*′, and the integration is now explicitly over *z* and *z*′. Changing the order of summation and integration, we obtain
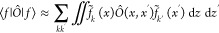
12Replacing *f̃*_*k*_ with its definition in eq 8 in terms of the vector of discrete function values at all grid point, *f*(***x***), then yields
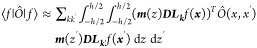
13Taking the discrete *f*(***x***), *f*(***x***′) out of the continuous double integral, we obtain
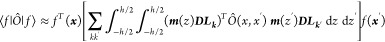
14From this we identify the discrete representation of the continuous operator *Ô*(*x*, *x*′) as
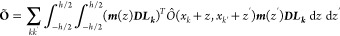
15such that (noting that *f*(***x***), *f*(***x***′) are the same as they refer to the function values at all grid points)
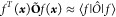
16Equation 15 is a general procedure for the construction of a discrete operator from a continuous operator consistent with FD. As a practical example, consider the one-dimensional time-independent Schrödinger equation:
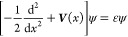
17With standard high-order FD, eq 17 would be solved by sampling ψ on a discrete grid, replacing d^2^/d*x*^2^ with its discrete counterpart, and representing the potential *V*(*x*) as a diagonal matrix with the external potential sampled on the discrete grid points. In our approach, we can use the definition in eq 15 with *V*(*x*, *x*′) = *V*(*x*)δ(*x* – *x*′) to build a potential operator that operates on ψ with a higher spatial sampling rate:
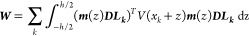
18where in going from eq 15 to eq 18 the integral over *z*′ vanishes owing to the Dirac delta function and the summation over *k*′ vanishes because from eq 8 we know that *f̃*_*k*_(*x*)*f̃*_*k*_′(*x*) = [*f̃*_*k*_(*x*)]^2^δ_*kk*′_ for any given *x*_*k*_. Equation 18 is exact in the limit of infinite grid and stencil. Practically, the integral over *z* around each grid point is approximated with a quadrature:
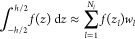
19using *N*_*l*_ evenly spaced sampling points with weights set to *w*_*l*_ = 1/*N*_*l*_. It is important to note that if we approximate the integral as only the value at the grid point, we recover the conventional FD. Using a denser quadrature allows for more accurate sampling of the potential, increasing numerical precision. The final expression for a discrete representation of the continuous multiplicative potential operator is then:
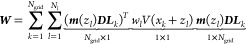
20where *V*(*x*_*k*_ + *z*_*l*_) is the potential sampled at the quadrature points *z*_*l*_ around grid point *x*_*k*_. Thus, whereas *V* is a local operator represented by a diagonal matrix, *W* is a semilocal operator owing to its use of the coefficient matrix *D*.

As an additional example of using eq 15, which is shown to be useful in the following section, consider a semilocal projection operator |**χ**(*x*)⟩ ⟨**χ**(*x*′)|, where the vector |**χ**(*x*)⟩ is a “short” vector, i.e., it has nonzero values only in some neighborhood around a given point. Inserting this operator in eq 15 yields
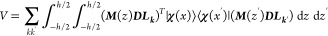
21Approximating the integral as a quadrature, if we define
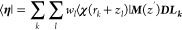
22then changing from the standard representation to the FDI representation simply entails using a modified projector, i.e.,
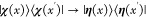
23Importantly, owing to the vector *L*_*k*_**η** only involves a few additional points as compared to **χ** and therefore the increase in the computational cost is minimal.

The generalization of the above considerations to 3D is straightforward: the stencil extends in all dimensions and the selection operator ***L***_*k*_ must be adjusted accordingly. There is a degree of freedom in selecting which stencil to use for interpolation in a general direction in 3D. The FD stencil can be designed to include mixed terms or higher order terms to improve the approximation.^54,59^ In this article, we use stencils composed of pure terms only, for simplicity.

## 3 Results and Discussion

### Model Potential

**Figure 2:**
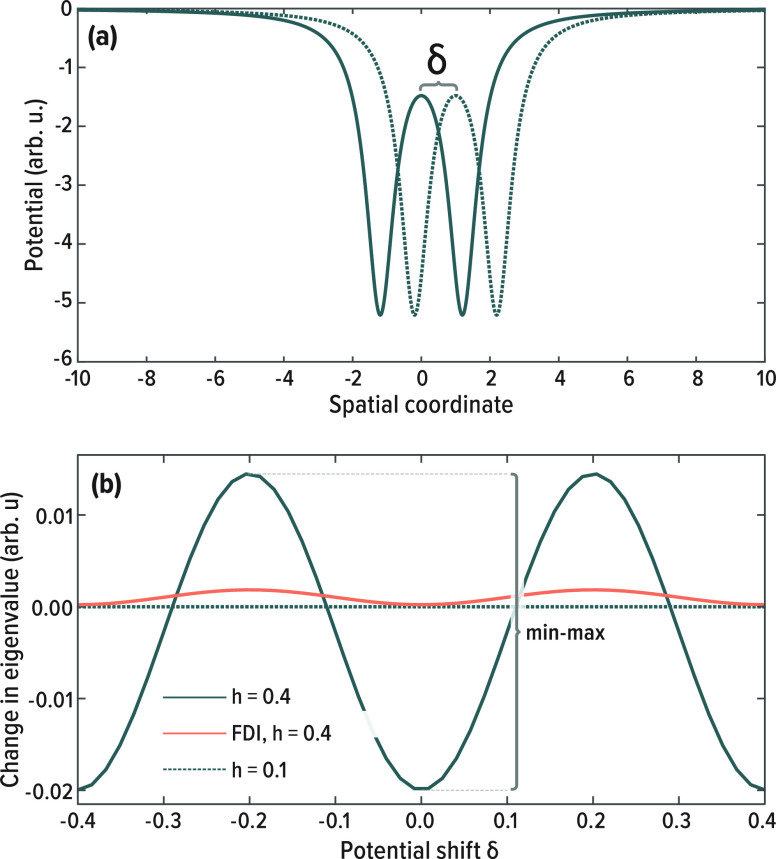
(a) Model potential defined in eq 24. Solid and dashed lines show the potential centered around zero and shifted by the parameter δ, respectively. (b) Deviation in the lowest eigenvalue, compared to a reference calculation with a grid spacing of 0.05, for a range of δ values using standard FD with grid spacing of 0.4 and 0.1 and using 12^th^ order FDI with a grid spacing of 0.4.

To illustrate the effect of introducing FDI we first consider the model system suggested by ref (56), a one-dimensional Schrödinger equation with a double-well Lorentzian potential, given by
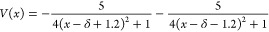
24The model potential is illustrated in Figure 2a. The parameter δ is introduced to shift the potential with respect to the grid. Figure 2b demonstrates the lowest eigenvalue calculated for the model system while sweeping the value of δ across the grid. We see that standard FD with a grid spacing of *h* = 0.4 yields a wide spread in the value of the first eigenvalue, whereas for the FDI calculation with *h* = 0.4, the spread is reduced by more than an order of magnitude. The standard calculation with *h* = 0.1 is converged to 7 digits, and its spread is no longer visible in the plot.

**Figure 3:**
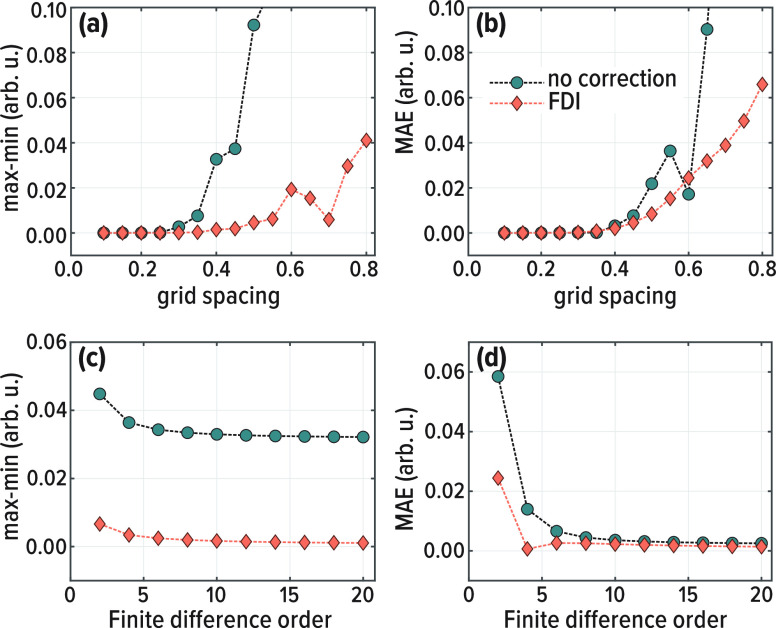
Summary of egg-box errors for the model potential of eq 24. Spread and MAE of the first eigenvalue, as a function of (a,b) grid spacing at the 12th order FD, (c,d) FD expansion order, using both standard FD and FDI, at a grid spacing of 0.4. As a reference for all calculations, we used the eigenvalue calculated with standard FD and a grid spacing of 0.05. The same FD expansion order was used for the Laplacian and FDI.

For quantitative analysis, we define the eigenvalue spread as the maximum difference in values it exhibits across the grid. We also assess the mean absolute error (MAE) of the eigenvalue, compared to a high precision reference solution with *h* = 0.05. The results, for the lowest eigenvalue, are illustrated in Figure 3. The figure clearly shows that both spread and MAE are reduced with increasing order of FDI. Already with a low-order FDI correction, there is significant improvement in spread that is not gained simply by increasing the FD expansion order per a given grid.

**Figure 4:**
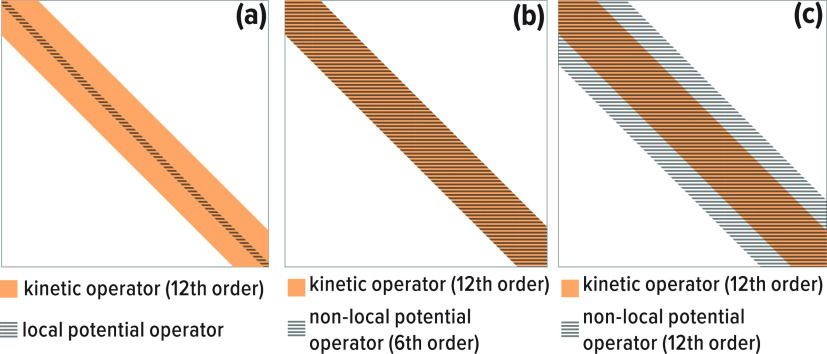
Sparse matrix patterns of operators in the model system, using a 12^th^ order FD kinetic energy operator, accompanied by (a) a local potential operator, (b,c) a 6^th^ and 12^th^ order, respectively, semilocal FDI operator.

The results presented in Figure 3 clearly show that FDI significantly improves the discrete representation of differential equations. However, the picture is not complete without a discussion of the associated computational cost. As mentioned above, large-scale sparse eigenvalue problems are generally treated with iterative solvers. The computational bottleneck in these solvers is in the sparse matrix-vector multiplication operations. The number of nonzero elements per row determines the cost of sparse vector-matrix multiplication. Therefore, as a measure of the computational cost, we compare the number of nonzero elements in the standard FD operators to the number of nonzero elements in the FDI operators. Figure 4 illustrates sparse matrix patterns of eq 17 using either the local operator *V* (i.e., standard FD) or the semilocal operator *W* of eq 20 (i.e., an FDI calculation). The semilocal operator width is double the width of the FD stencil from which it is built. However, in the context of Schrödinger-like equations with a FD nonlocal kinetic energy operator, there is overlap between the semilocal potential operator and the kinetic energy operator, substantially decreasing the additional cost FDI introduces on top of the standard FD. This should be compared to the generally steeper cost of using a finer grid, where at least linear scaling with respect to the grid size is expected.

### DFT Calculations

We now turn to the application of FDI to DFT calculations. Although in such calculations all potential terms may introduce egg-box effects, it is known that the majority of the egg-box error arises from the pseudopotential (PP) which represents the electron–ion attraction.^54,56^ Therefore, we apply FDI only to the pseudopotential operators.

In the case of a norm-conserving PP, which is of standard use in real-space DFT calculations,^43^ the PP of a given atom is in principle a nonlocal operator that operates separately for each angular momentum channel. Naively, application of the PP requires semilocal projectors of the form.
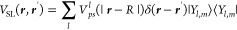
25where *V*_*ps*_^*l*^ is the PP corresponding to the *l*^th^ angular momentum, *R* is the coordinate of the atom, and *Y*_*l*,*m*_(θ, ϕ) is the *lm*^th^ spherical harmonic. However, evaluating ⟨ϕ|*V*_*ps*_(*r*, *r*′)|ϕ⟩ on a real space grid results in a dense matrix. To avoid this dense representation, the projection scheme suggested by Kleinman and Bylander^60^ (originally in the context of planewave calculations) is commonly used to represent eq 25 as the sum of a multiplicative potential and a weighted sum of projector operators, in the form:^26^
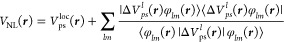
26where *V*_ps_^loc^(***r***) is an arbitrary component chosen as the local PP, Δ*V*_ps_^*l*^(***r***) ≡ *V*_ps_^*l*^(***r***) – *V*_ps_^loc^(***r***), and φ_*lm*_(***r***) is the pseudo-wave-function with *lm* quantum angular momentum numbers. The projection scheme is never constructed explicitly. Instead, we note that |Δ*V*_ps_^*l*^φ_*lm*_⟩ is a “short” vector around each atom, because PPs with different *l* only differ inside a small core region. Therefore, the FD and FDI representations of the semilocal components are obtained using the left-hand and right-hand sides of eq 23, respectively. The FDI representation of the local part of the PP is simply given by eq 20 as above.

Before proceeding to show numerical results, we note that estimating the computational cost increase in a 3D DFT calculation is similar in spirit to that given above for the 1D model, but is more complex. First, the FDI matrix representation of the Hamiltonian contains more nonzero entries per row than in the 1D case, leading to increased communication needs across parallel regions. The extent of this generally depends heavily on practical details of the implementation, including hardware specifics, the system being calculated, and the parallelization paradigm of the real-space grid. Second, the use of a coarser grid, enabled by the FDI approach, generally leads to a more compact eigenvalue spectrum than the corresponding FD calculation with a finer grid. This can be advantageous for iterative eigensolvers used in real-space calculations and may benefit the overall convergence of the self-consistent loop, requiring fewer iterations to converge.

Turning to illustrative examples, we choose a hydrogen atom moving relative to the grid, a methane molecule rotating with respect to the grid, and the dissociation curve of methane. These examples represent three different scenarios in which egg-box errors are commonly manifested. All calculations were performed within the PARSEC (pseudopotential algorithm for real space electronic-structure calculations) suite,^36,44^ in which we have implement the FDI representation. For simplicity, all calculations were performed using the local density approximation (LDA) and Troullier-Martins^53^ PPs with cutoff radii of 1.18 Bohr and 1.08 Bohr for hydrogen and carbon, respectively. We do not expect calculations within the generalized-gradient approximation (GGA) to behave differently as we find the egg-box effect to be dominated by the electron–ion interaction.

**Figure 5:**
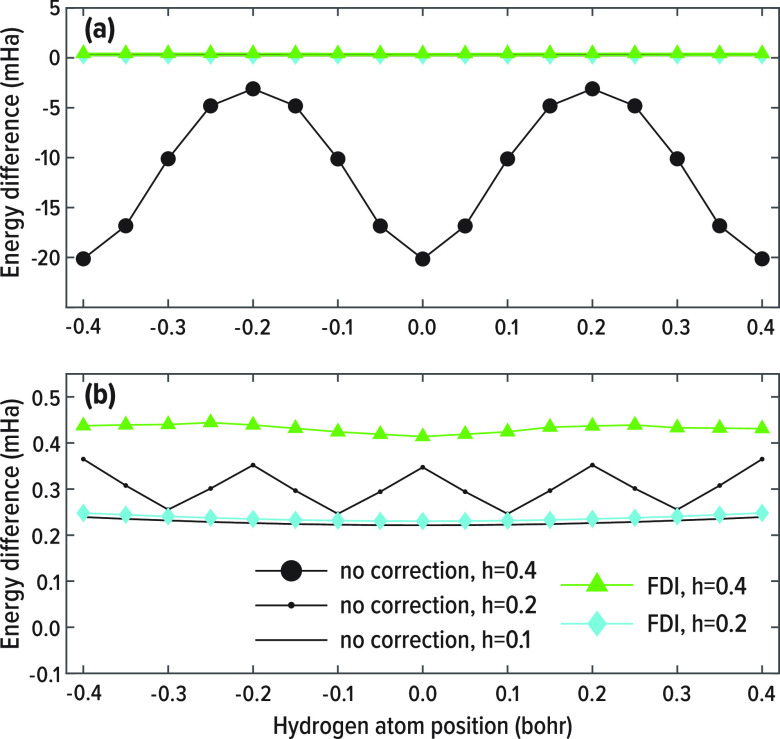
Difference of the total energy of a hydrogen atom, calculated within the LDA using standard FD at *h* = 0.4, 0.2, 0.1, and 18th order FDI at *h* = 0.4, 0.2, with respect to a reference calculation with a high-density radial grid. The *x*-axis represents the positions of the hydrogen atom with respect to the real space grid. Panel (b) is zoomed in from panel (a).

Figure 5 shows the change in energy of a hydrogen atom upon translation along the grid, with respect to a high-precision reference calculation performed using a radial grid. At a large grid spacing, *h* = 0.4, the egg-box error is on the order of tens of mHa. Using FDI at the large grid spacing *h* = 0.4, the egg-box error is reduced by 3 orders of magnitude. Furthermore, while the error is diminished for a finer grid spacing of 0.2, using FDI with *h* = 0.2 provides further improvement and yields a solution close to that obtained from a standard FD at *h* = 0.1, i.e., with a grid eight times as dense.

**Figure 6:**
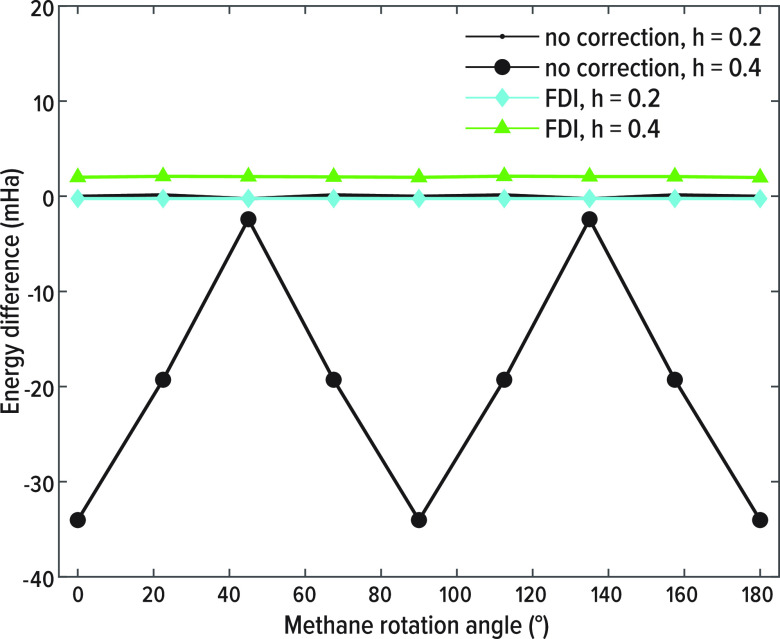
Difference of the total energy of a methane molecule calculated within the LDA using standard FD and 18th order FDI at *h* = 0.4, 0.2, as a function of the methane angle relative to the grid. The difference is with respect to a reference calculation of methane at a grid spacing of *h* = 0.1.

Rotation of a methane molecule is another rigorous test, because the four hydrogen atoms of the molecule cannot all be aligned with the grid simultaneously and nonetheless need to be treated equally. The results are shown in Figure 6, which again shows that the FDI representation reduces the egg-box errors—at *h* = 0.4 from tens of mHa in the absence of FDI to the order of 10^–3^ mHa with an 18th order FDI.

**Figure 7:**
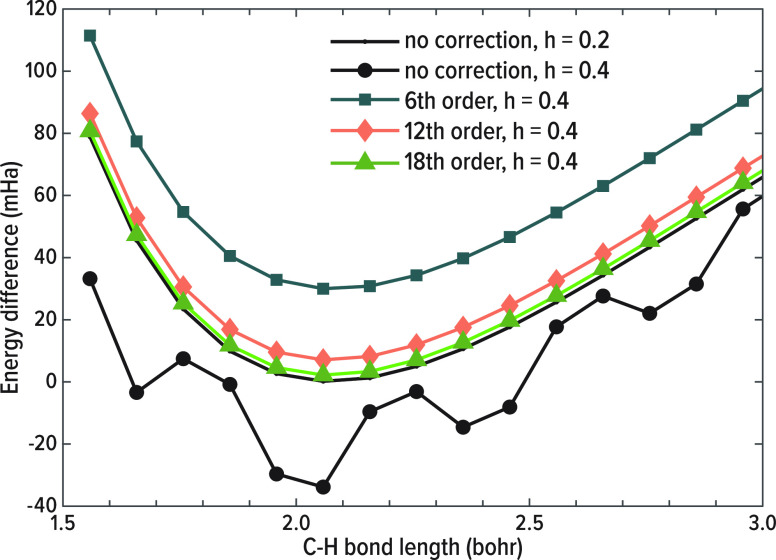
Methane dissociation curves, calculated within the LDA using standard FD at *h* = 0.4, 0.2 and 6th, 12th, and 18th order FDI at *h* = 0.4, as a function of the *z*-axis aligned C–H bond length. The dissociation curves are presented as the difference of the total energy with respect to a reference calculation of methane at its equilibrium geometry with a fine grid spacing of *h* = 0.1.

**Figure 8:**
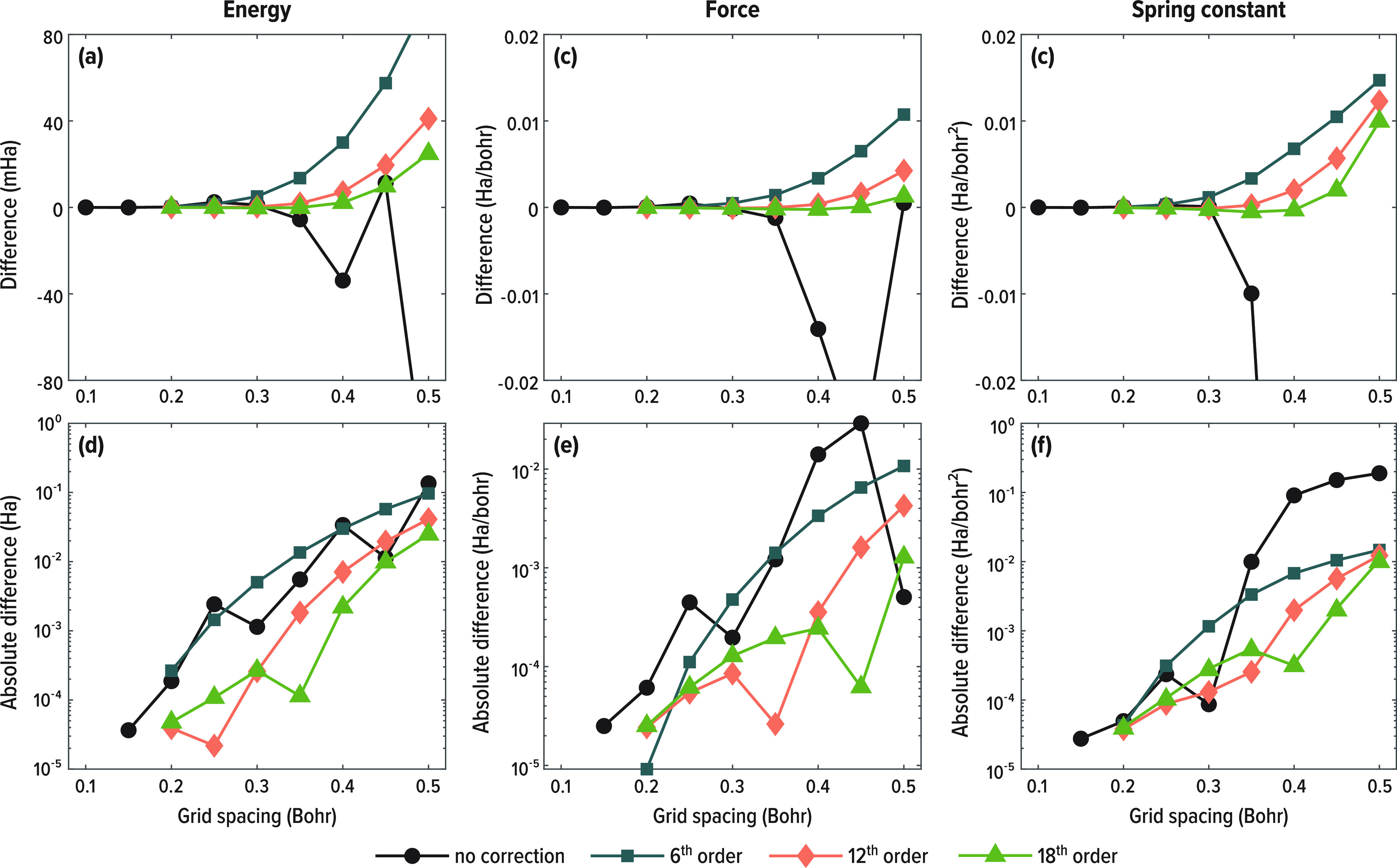
Convergence of methane-related quantities at the equilibrium bond length as a function of grid spacing, with standard FD and with FDI, all given as differences with respect to a calculation at a grid spacing of 0.1. (a) Energy, (b) force on one of the H atoms, (c) “spring constant” (second derivative) associated with movement of the same H atom. (d)-(f) Log-scale absolute errors plots corresponding to the data in panels (a)-(c).

Next, we examined the dissociation curve of one hydrogen atom from the methane molecule, obtained by moving one of the hydrogen atoms away from its equilibrium position along the C–H bond direction. The results are summarized in Figure 7. Clearly, at the coarse grid spacing of *h* = 0.4, standard finite difference calculations yield a nonsmooth and chemically nonsensical curve, which would lead to catastrophic errors in applications such as structural relaxation or molecular dynamics. Conversely, FDI recovers the expected smooth behavior. The figure also demonstrates how the FD stencil expansion order used for the FDI affects the results: already with a minimal sixth order expansion, smoothed curves are obtained. Using higher expansion orders yields even better precision. Finally, Figure 8 summarizes the trends obtained from the methane dissociation example, showing that indeed the FDI representation improves not only the precision of energy calculation, but also the precision of its numerically sensitive derivative quantities, namely force (first derivative) and spring constant (second derivative) calculations.

Taken together, the above results firmly establish that using FDI allows us to perform simulations at substantially larger grid spacings without sacrificing numerical precision.

## 4 Conclusions

In this article, we addressed the limitations of the standard finite difference method, manifested by egg-box errors upon translation and/or rotation with respect to the underlying numerical grid. Such errors are of particularly severe consequences in the context of, e.g., geometry relaxation, vibrational frequency calculations, and molecular dynamics, where the nonphysical undulation of the energy may lead to qualitative failures in the evaluation of forces and spring constants, unless a fine grid spacing is used.

We presented a finite difference interpolation scheme for representing potential operators on the grid and showed that it mitigates egg-box errors significantly for both model potential calculations and practical DFT calculations. This opens the door to higher precision DFT calculations across a wide range of scenarios using a relatively coarse grid, therefore accelerating and even outright enabling large-scale calculations, especially if used in conjunction with judiciously chosen pseudopotentials. We plan to explore this issue in future work.

## Appendix A: Deriving Finite Difference Coefficients

A finite difference stencil is composed of a set of neighbors around the point of interest, and a numerical coefficient for each point. Here we show practical recipes for finding the FD coefficients.

We start with an explicit example of solving for the FD coefficients of a 5 point stencil. Given a 1D function *f*(*x*) defined on a grid with grid spacing *h*. The function values at two neighboring grid points in each direction can be expanded as fourth order Taylor series.

A1

The above eqs A1 can be thought of as a set of equations for the four derivatives , *i* = 1, 2, 3, 4, in terms of the values of *f*() at *x* and neighboring grid points *x* ± *h* and *x* ± 2h. For example, the first two derivatives turn out to be

A2

A3

These equations immediately lead to

A4

where the matrix denoted as ***D*** is exactly the one defined in eq 6 for this particular order of expansion.

The FD stencils can also be found in a procedural manner, based on the inversion of the Vandermonde (design) matrix that recovers traditional finite difference stencils. The advantage of this procedure is that it can be used for any dimension of stencil and any arrangement of neighboring points. To illustrate it, consider the same 5-point FD stencil for a 1D function *f*(*x*) as above. Approximating the function by a fourth order polynomial, we have

A5

the function derivatives at *x* = 0 will be

A6

To solve for the polynomial coefficients we build an equation set for the five points: Arranging the powers using a Vandermonde (design) matrix, ***M***, we obtain

A7

such that

A8

If the points are chosen as

A9

we obtain

A10

A11

The derivative coefficient matrix ***D*** is then obtained from ***M***^–1^ by multiplying the *n*^th^ row of this matrix by a (*n* – 1)! prefactor. Indeed, for this case ***D*** of eq A4 is immediately recovered.
